# Supplementary material for: COZOID: contact zone identifier for visual analysis of protein-protein interactions
Source: BMC Bioinformatics. 2018 Apr 6;19:125. doi: 10.1186/s12859-018-2113-6 (PMC5889581; doi:10.1186/s12859-018-2113-6)
Supplement: Supplementary file 2 — Software build. Executable binary file of the software tool. (ZIP 118,784 kb) [file 12859_2018_2113_MOESM2_ESM.zip › cozoid/java/docs/beansbinding-1.2.1-doc.zip!/index-files/index-14.html]

N-Index (Beans Binding)


---


|  |  |  |
| --- | --- | --- |
| **Overview** \| Package \| Class \| Use \| **Tree** \| **Deprecated** \| **Index** \| **Help** | |  |
| **PREV LETTER**   **NEXT LETTER** | **FRAMES**    **NO FRAMES**     **All Classes** |  |


A B C D E F G H I J K L M N O P Q R S T U V W 

---


## **N**

**name** - Variable in class org.jdesktop.el.impl.lang.FunctionMapperImpl.Function: **NE0** - Static variable in interface org.jdesktop.el.impl.parser.ELParserConstants: **NE1** - Static variable in interface org.jdesktop.el.impl.parser.ELParserConstants: **newToken(int)** - Static method in class org.jdesktop.el.impl.parser.Token: Returns a new Token object, by default. **next** - Variable in class org.jdesktop.el.impl.parser.Token: A reference to the next regular (non-special) token from the input stream. **Node** - Interface in org.jdesktop.el.impl.parser: **NodeVisitor** - Interface in org.jdesktop.el.impl.parser: **NonLiteral()** - Method in class org.jdesktop.el.impl.parser.ELParser: **NOT0** - Static variable in interface org.jdesktop.el.impl.parser.ELParserConstants: **NOT1** - Static variable in interface org.jdesktop.el.impl.parser.ELParserConstants: **notifySynced()** - Method in class org.jdesktop.beansbinding.Binding: Notifies all registered `BindingListeners` of a successful sync (`refresh` or `save`), by calling `synced` on each one. **notifySyncFailed(Binding.SyncFailure)** - Method in class org.jdesktop.beansbinding.Binding: Notifies all registered `BindingListeners` of a failure to sync (`refresh` or `save`), by calling `syncFailed` on each one. **Null()** - Method in class org.jdesktop.el.impl.parser.ELParser: **NULL** - Static variable in interface org.jdesktop.el.impl.parser.ELParserConstants

---


|  |  |  |
| --- | --- | --- |
| **Overview** \| Package \| Class \| Use \| **Tree** \| **Deprecated** \| **Index** \| **Help** | |  |
| **PREV LETTER**   **NEXT LETTER** | **FRAMES**    **NO FRAMES**     **All Classes** |  |


A B C D E F G H I J K L M N O P Q R S T U V W 

---
